# Pheochromocytoma Across the Perioperative Course: Current Concepts and Clinical Management

**DOI:** 10.1155/anrp/4406992

**Published:** 2026-09-29

**Authors:** Aibek E. Mirrakhimov, Nicole Bashall

**Affiliations:** ^1^ Department of Anesthesiology, Perioperative, Critical Care, and Pain Medicine, Division of Adult Cardiothoracic Anesthesiology, University of Kentucky, Lexington, Kentucky, USA, uky.edu; ^2^ Division of Adult Cardiothoracic Anesthesia and Intensive Care and Resuscitation, Cleveland Clinic Foundation, Cleveland, Ohio, USA, clevelandclinic.org

## Abstract

Pheochromocytoma is an uncommon neuroendocrine tumor with myriad nonspecific clinical presentations. If not recognized and treated, it is associated with the development of target organ damage and complications such as myocarditis, cardiomyopathy, cerebrovascular accidents, and diabetes mellitus, among numerous others. The pheochromocytoma diagnosis is based on biochemical proof of catecholamine excess followed by imaging localization of the tumor. Patients with newly diagnosed pheochromocytoma should be seen by a multidisciplinary perioperative team, including the anesthesiologist, operating surgeon, endocrinologist, and cardiologist. Perioperative antihypertensive therapy should be instituted prior to adrenal resection to allow for cardiovascular recovery and to hopefully mitigate the hypertensive response to laryngoscopy and tumor manipulation. The anesthesiologist must be prepared to manage both intraoperative hypertension, hypotension, and glycemic disturbances, among other possible complications. Patients with previously undiagnosed pheochromocytoma who present for unrelated surgery represent a high‐risk group. Once intraoperatively suspected, priority should be given to immediate stabilization, cessation of surgery when feasible, and close communication with the surgical team regarding whether the procedure should be aborted, limited, or continued based on the urgency of the operation and the patient’s clinical status. In most cases, nonessential surgery should be aborted. The postoperative level of care should be based on intraoperative patient courses and institutional protocols and guidelines to monitor and manage postresection hypoglycemia and vasoplegia, when they occur. This narrative review aims to provide practicing anesthesiologists with an updated, practical framework for the perioperative recognition and management of pheochromocytoma. We integrate contemporary evidence with anesthesia‐specific decision‐making, including the management of catecholamine‐induced cardiovascular dysfunction, perioperative fluid and vasoactive therapy, medication‐triggered crises, and a dedicated approach to previously undiagnosed pheochromocytoma presenting during unrelated surgery.

## 1. Introduction

Pheochromocytoma represents a rare tumor stemming from the adrenal medulla [1], whereas paragangliomas originate from extra‐adrenal chromaffin cells [2]. Despite being uncommon and advances in timely diagnosis and management, pheochromocytoma is still associated with increased perioperative morbidity and mortality though better than historical cohorts [3, 4], which is especially true for patients with undiagnosed pheochromocytoma presenting for surgery [5]. On a functional level, pheochromocytomas and paragangliomas can be biochemically silent [2, 6] or secrete (either episodically or continuously) various catecholamine hormones (such as dopamine, norepinephrine, and epinephrine) [2, 7].

Unfortunately, late diagnosis of pheochromocytoma is common due to protean and nonspecific clinical presentation. It has been named the great masquerader by some [8]. Because of the late diagnosis, some patients develop complications such as cardiomyopathy, acute myocarditis, and cerebrovascular accidents, to name a few [9]. Therefore, some patients afflicted with pheochromocytoma may present to the care of anesthesiologists in a decompensated state [10].

We aim to provide a contemporary and practical resource for perioperative management of pheochromocytoma for practicing anesthesiologists. First, we will cover the foundational basics of adrenal embryology, anatomy, and relevant biochemistry of the adrenal medulla. Second, we will review the epidemiology of pheochromocytoma, followed by a clinical presentation and diagnostic approach for patients suspected of having pheochromocytoma. Finally, we will discuss preoperative optimization, intraoperative considerations, postoperative concerns, and management. The current review focuses on pheochromocytoma, but many of the aspects described apply to the anesthetic concerns of patients with paraganglioma.

## 2. Basics of Adrenal Embryology, Anatomy, and Adrenal Medulla Biochemistry

The adrenal gland is a small paired retroperitoneal organ initially described by Italian anatomist Eustachius [11]. Adrenal glands are positioned on the superior pole of each kidney, which is why they are also known as suprarenal glands. Arterial blood supply to the adrenal gland is provided by three arteries—the superior, middle, and inferior adrenal arteries. The adrenal vein provides sole venous drainage; however, pheochromocytomas exhibit angiogenesis, with the development of additional pathologic vascularity, adding to surgical challenges. It is interesting to note that the right adrenal vein drains directly into the inferior vena cava, whereas the left adrenal vein drains into the left renal vein [12].

On a histological level, the adrenal gland consists of the adrenal cortex and adrenal medulla [13]. The adrenal cortex is further subdivided into zona glomerulosa, predominantly synthesizing aldosterone; zona fasciculate, synthesizing cortisol; and the zona reticularis, which synthesizes estrogens. The adrenal medulla, which is the innermost part of the gland, is responsible for producing catecholamines. This distinct anatomical and histological composition of the adrenal gland stems from different embryological origins. For example, the adrenal cortex and kidney share the exact embryological origin, the mesoderm. In contrast, the adrenal medulla stems from neural crest cells that migrated from the celiac sympathetic plexus [14]. In contrast, paragangliomas originate from extra‐adrenal neural crest cells [15].

The adrenal medulla and extra‐adrenal paraganglia are responsible for the synthesis and secretion of catecholamines [16]. Catecholamine hormones play an essential role in regulating numerous vital functions such as cardiovascular performance, kidney function, insulin secretion, and the central nervous system [16]. The precursor of catecholamines is the amino acid tyrosine [17], and the simplified graph of catecholamine synthesis and metabolism is presented in Figures 1 and 2, respectively. Catecholamines exert their physiological effects via various cellular receptors [16]. A summary of receptors and their effects is presented in Table 1.

**FIGURE 1:**
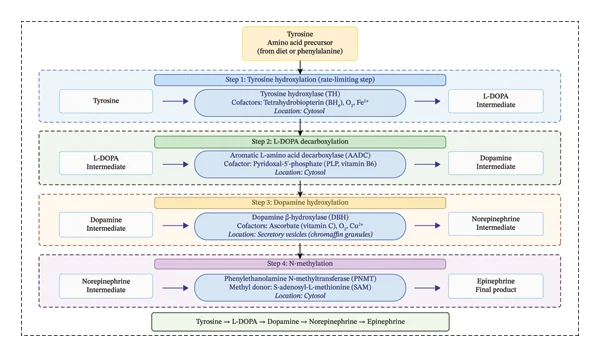
Catecholamine synthesis.

**FIGURE 2:**
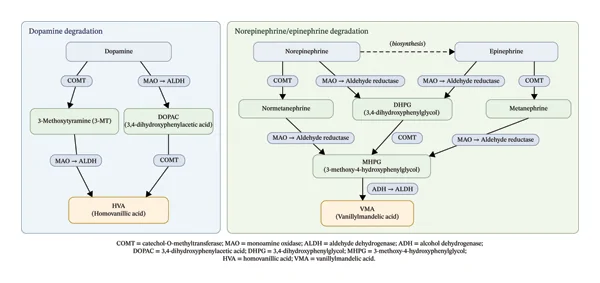
Catecholamine metabolism.

**TABLE 1:** Catecholamine receptors and their physiological effects.

Receptor	Location	Stimulation effects
α‐1	Smooth muscle	Smooth muscle contraction, gluconeogenesis
α‐2	Presynaptic terminals, platelets, pancreatic islet cells, smooth muscle	Inhibition of neurotransmitter release, vasoconstriction, platelet aggregation, decreased insulin secretion
β‐1	Heart, kidney	Increase in heart rate, cardiac contractility, and renin release
β‐2	Smooth muscle, liver, bronchioles	Smooth muscle relaxation, potentiation of insulin release
Dopamine‐1	Renal, mesenteric, cerebral, and coronary circulation	Vasodilation, diuresis, and natriuresis
Dopamine‐2	Presynaptic sympathetic nerve terminals, central nervous system, anterior pituitary, kidney, adrenal cortex	Inhibition of norepinephrine/dopamine release, inhibition of prolactin secretion, potentiation of renal sodium, and water excretion

## 3. Epidemiology of Pheochromocytoma

Pheochromocytoma is a rare clinical entity. However, the incidence is rising in part due to improved diagnosis. For instance, Berends et al. studied the pathology registry in the Netherlands from 1995 until 2015 [18]. They have demonstrated that the age‐adjusted incidence of pheochromocytoma increased from 0.29 to 0.46 per 100,000 patients. In a similar study from Denmark, Ebbehoj et al. performed a retrospective cohort analysis of patients diagnosed with pheochromocytoma from 1977 until 2015 [19]. Ebbehoj et al. found that the age‐standardized incidence of pheochromocytoma increased from 1.4 to 6.6 per million person‐years.

Ebbehoj and colleagues also conducted another retrospective cohort study in Olmsted County, Minnesota [20]. The study included data from 1997 until 2017 and demonstrated that the standardized age incidence rates increased from 4.4. to 47 [8]. The prevalence of pheochromocytoma in patients with hypertension appears to be ranging from 0.2% to 0.6% [21]. Of note, the prevalence of pheochromocytoma in patients with resistant hypertension is much higher and may reach up to 4% [22]. Pheochromocytoma is a common diagnosis among patients with adrenal tumors, such as in a study by Chandrasekar et al., where it represented 37.5% out of 4695 patients with adrenal tumors [23].

## 4. Clinical Presentation of Pheochromocytoma

The clinical presentation of pheochromocytoma is highly variable and ranges from incidental or minimally symptomatic disease to multiorgan failure and shock [8, 24, 25]. Clinical manifestations may reflect catecholamine excess, tumor size, or local mass effect [24]. In addition to classic adrenergic features, patients may present with metabolic, cardiovascular, and cerebrovascular complications, including diabetes mellitus [26], cardiovascular injury [27], and cerebrovascular events [28].

Pheochromocytoma has historically been described by the “rule of 10,” which states that approximately 10% of tumors are malignant, 10% are bilateral, 10% are extra‐adrenal, and 10% are familial [29]. Although this construct remains a useful historical teaching aid, it oversimplifies the disease spectrum, particularly in the era of contemporary imaging and genetic testing. Patients with familial syndromes associated with pheochromocytoma may also exhibit additional manifestations related to the underlying disorder [29, 30]. A brief overview of familial syndromes associated with pheochromocytoma is presented in Table 2.

**TABLE 2:** Genetic syndromes associated with pheochromocytoma.

Syndrome	Mode of inheritance	Risk of pheochromocytoma	Associated conditions
Von‐Hippel Landau	Autosomal dominant	10%–20%	Hemangioblastoma (cerebellum, spinal cord, brainstem), retinal angioma, clear cell renal cancer, pancreatic tumors
Multiple endocrine neoplasia (MEN) Type 2a	Autosomal dominant	50%	Medullar thyroid cancer, parathyroid hyperplasia
Multiple endocrine neoplasia (MEN) Type 2b	Autosomal dominant	50%	Medullary thyroid cancer, mucocutaneous fibromas, Marfanoid habitus
Neurofibromatosis Type 1	Autosomal dominant	3%	Neurofibromas, gliomas, bony abnormalities
Carney–Stratakis dyad	Autosomal dominant	Up to 73%	Gastrointestinal stromal tumors

The classic symptom triad consists of episodic headache, sweating, and tachycardia [31]. However, contemporary data suggest that this triad is absent in most patients. Baguet et al. reported that only 24% of patients with pheochromocytoma exhibited all 3 classic symptoms [32]. When considered individually, these manifestations remain common. Headache of varying severity has been reported in up to 90% of patients, and elevated blood pressure, whether sustained or paroxysmal, occurs in approximately 95% [33]. The episodic nature of symptoms usually reflects intermittent catecholamine release, which may occur spontaneously or be triggered, such as during physical examination with tumor manipulation or in a pregnant patient lying supine when the uterus compresses the tumor [34]. Orthostatic hypotension and syncope may also occur and likely reflect chronic vasoconstriction with relative intravascular volume depletion together with adrenergic receptor desensitization in the setting of catecholamine excess [31].

Cardiovascular manifestations are among the most important and potentially severe clinical features of pheochromocytoma. Perioperative myocardial events and cerebrovascular accident occurred in 3.7% of patients, and postoperative intensive care unit admission occurred in 13.6% [24]. From a mechanistic standpoint, cardiovascular injury in pheochromocytoma is often not driven by acute coronary occlusion, but rather by catecholamine‐mediated myocardial injury. Manifestations include myocardial stunning, demand ischemia, myocarditis, and cardiomyopathy [27]. The associated ventricular dysfunction is frequently reversible and may appear as dilated cardiomyopathy, Takotsubo‐pattern cardiomyopathy, or, less commonly, a hypertrophic phenotype [27]. Reported Takotsubo variants include the classic apical, reverse, and midventricular forms [27]. Scholten et al. reported a case of previously undiagnosed pheochromocytoma presenting with decompensated systolic heart failure and ventricular tachycardia [35]. These cardiac presentations may closely mimic acute coronary syndrome because patients may present with chest pain, ischemic electrocardiographic changes, troponin elevation, regional wall motion abnormalities, and even cardiogenic shock [27]. In many such cases, however, the dominant mechanism is catecholamine‐mediated myocardial toxicity, microvascular dysfunction, or stress cardiomyopathy rather than acute epicardial coronary occlusion [27]. This distinction is clinically important because myocardial dysfunction is expected to improve substantially after hemodynamic stabilization and adrenergic blockade, before definitive tumor resection [27].

When pheochromocytoma presents with acute myocarditis or cardiomyopathy, the timing of adrenalectomy should be individualized. In most patients, it is reasonable to allow recovery of ventricular function and end‐organ injury before proceeding with resection while initiating alpha‐adrenergic blockade and volume optimization. Selected patients may require temporary mechanical circulatory support as a bridge to recovery and later surgery. Emergency tumor resection is rarely the preferred approach and results in poorer outcomes [35]. At present, there are no high‐quality prospective data defining the optimal waiting period after pheochromocytoma‐related myocarditis or Takotsubo syndrome; the available literature consists largely of retrospective studies, case series, and case reports [36–40]. Therefore, pending robust research data, the timing of surgery should be guided by the clinical trajectory, recovery of cardiac function, resolution of end‐organ injury, and multidisciplinary consensus. Not all manifestations of pheochromocytoma are attributable to excess of catecholamines. In some cases, clinical findings may instead be related to tumor size, local mass effect, or compression of adjacent structures [24]. Rarely, local tumor extension may occur, including involvement of the inferior vena cava by tumor thrombus [41].

Pheochromocytoma is a great mimicker and may occasionally present with nonspecific syndromes rather than classic adrenergic symptoms [1, 2, 8, 26]. Reported atypical presentations include fever of unknown origin, chest/abdominal pain, cardiogenic shock, new‐onset psychiatric manifestations such as anxiety, panic‐like episodes, insomnia, or rarely psychosis, and new‐onset diabetes mellitus or otherwise unexplained hyperglycemia. These findings likely reflect catecholamine excess, associated cytokine release in selected cases, and metabolic dysregulation with impaired insulin secretion and increased insulin resistance. Other misleading presentations include weight loss, gastrointestinal dysmotility, cardiomyopathy, arrhythmias, pulmonary edema, and stroke‐like neurologic syndromes. Accordingly, pheochromocytoma should remain in the differential diagnosis when otherwise unexplained constitutional, psychiatric, metabolic, and cardiovascular abnormalities occur together, particularly in the setting of labile or paroxysmal hypertension. In addition, the diagnosis should be considered with a hypertensive event after administration of metoclopramide, droperidol, or glucocorticoids, drugs which can all trigger a pheochromocytoma crisis.

## 5. Diagnostic Approach to Patients With Suspected Pheochromocytoma

The Endocrine Society comprehensive guideline on pheochromocytoma was last published in 2014 [42]. Testing for pheochromocytoma should be considered for patients with adrenal incidentalomas, signs and symptoms suggestive of excess catecholamines (such as paroxysmal tachycardia and hypertension), and a previous history of pheochromocytoma and in patients with hereditary syndromes associated with it. It is best to coordinate this testing with the patient’s endocrinologist.

The initial test should be either a free plasma metanephrines test or a 24‐h urine fractionated metanephrines collection rather than a vanillylmandelic acid test due to its higher sensitivity and specificity [7, 17, 31, 42]. The Endocrine Society guideline does not endorse whether free plasma metanephrines or 24‐h urine fractionated metanephrines collection should be the test of choice or whether they should be combined. Both tests have an excellent sensitivity of more than 95%, although the specificity of the urine test is higher, at approximately 98%, compared to around 90% for plasma‐free metanephrines, but the turnaround time for the blood test is shorter.

It is important to recognize that metanephrine and normetanephrine are metabolically produced derivatives of catecholamines and are physiologically inactive. Their elevation primarily reflects tumor metabolic activity rather than the magnitude of circulating catecholamine effects. Since pheochromocytomas contain catechol‐O‐methyltransferase (COMT), a portion of the catecholamines produced by the tumor may be converted to metanephrines within the tumor before entering the systemic circulation. As a result, elevated metanephrine concentrations do not necessarily correspond directly to circulating free catecholamine levels or the physiological activity of the tumor.

Measurement of 24‐h urinary free catecholamines (epinephrine and norepinephrine) may therefore provide complementary information regarding the physiological catecholamine output of the tumor and can be helpful in selected clinical scenarios. This may be valuable in some patients whose symptoms are related to other conditions beside pheochromocytoma.

It is important to note that urine creatinine should also be measured to ensure adequate urine collection was achieved if a 24‐h urine fractionated metanephrines or free catecholamines collection test was chosen [42]. The plasma‐free metanephrine test should be collected in a supine position after at least a 30‐min rest period [42].

If possible, it is recommended to discontinue medications that can lead to erroneous results for at least 2 weeks before the test. These medications can either impact the method of measurement or affect the metabolism of catecholamines in vivo [42]. Some of these interfering medications include tricyclic antidepressants, cyclobenzaprine, levodopa, antipsychotic agents, and sympathomimetics (e.g., cocaine, amphetamines). In patients with borderline results, initial biochemical assays can be resampled if the initial collection was likely suboptimal (e.g., not performed in a supine and properly rested person prior to plasma testing) or an additional laboratory test obtained. Some of the supplementary chemical assays that can be used include serum chromogranin A and clonidine suppression tests [42].

Protocolized adrenal imaging is recommended when initial biochemical testing is positive or when clinical suspicion remains despite equivocal results. Computed tomography (CT) and magnetic resonance imaging (MRI) are both reasonable first‐line imaging modalities, provided there are no contraindications [43]. Each modality has relative advantages, but both offer good diagnostic sensitivity and specificity. Imaging features that may suggest pheochromocytoma include a hypervascular adrenal mass, hemorrhagic or cystic change, attenuation greater than 10 Hounsfield units (usually 30–40) on noncontrast CT, and delayed contrast washout [39]. Available data suggest that administration of low‐osmolar [44] and nonionic [45] contrast agents is generally safe in patients with pheochromocytoma and does not appear to provoke clinically significant catecholamine release. Gallium‐68 DOTATATE PET/CT should be considered when clinical suspicion remains high despite negative initial imaging or metastatic disease. Representative CT and MRI appearances of pathologically confirmed pheochromocytoma are shown in Figures 3 and 4.

**FIGURE 3:**
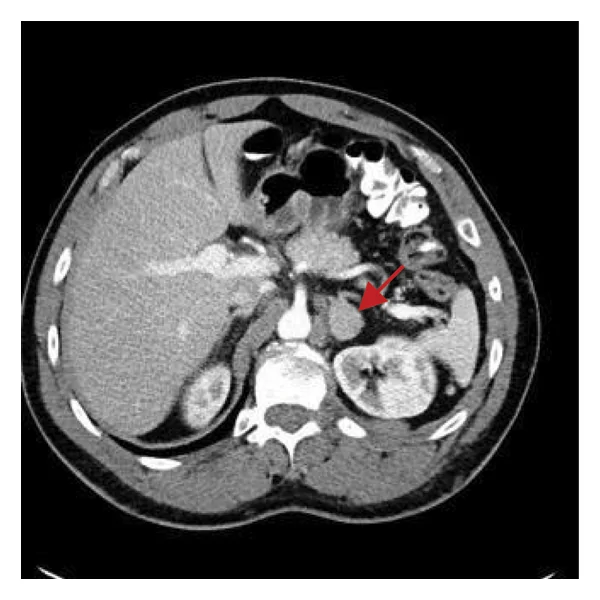
CT scan demonstrating a left‐sided adrenal lesion (red arrow) proven to be pheochromocytoma.

**FIGURE 4:**
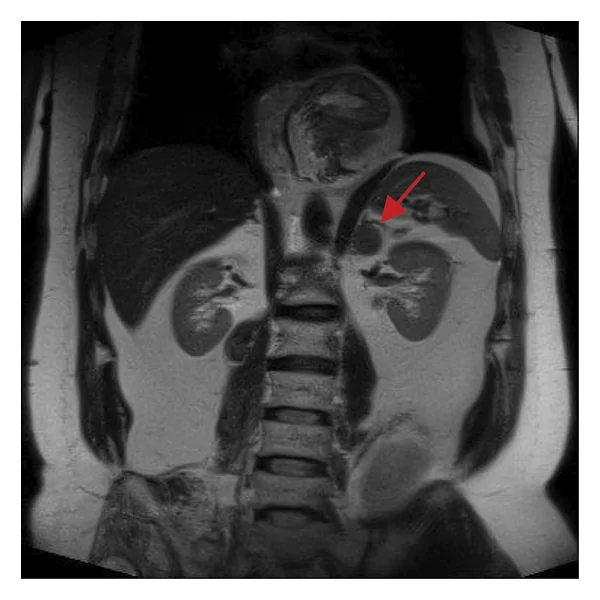
MRI scan demonstrating a left‐sided adrenal lesion (red arrow) proven to be pheochromocytoma.

### 5.1. Treatment Options and Preoperative Optimization

All patients with pheochromocytoma should undergo formal preoperative anesthesia evaluation before surgery. Ideally, pharmacologic preparation should be initiated 7–14 days before adrenalectomy, and many centers aim for at least 10 days of optimization when feasible, particularly in patients with hormonally functional tumors [42, 46]. The goals of adrenergic blockade is not primarily limited to attenuation of intraoperative hemodynamic instability, but rather includes resolution of episodic hypertensive events and associated symptoms, reversal of chronic catecholamine‐mediated vasoconstriction, correction of relative intravascular volume contraction, and recovery of end‐organ injury, particularly pheochromocytoma‐related cardiomyopathy. All this is expected before tumor resection. In clinical practice, selective alpha‐1 adrenergic blockade is usually initiated at a low dose and gradually up‐titrated every 3–4 days over several weeks until adequate hemodynamic control and evidence of physiologic blockade are achieved [33]. Dose escalation is typically guided by blood pressure response, the development of mild orthostatic hypotension, and attainment of recommended hemodynamic targets. Transient orthostatic tachycardia is common after initiation of alpha‐adrenergic blockade and does not necessarily indicate inadequate preparation, whereas persistent resting tachycardia may suggest incomplete blockade or ongoing catecholamine excess requiring further pharmacologic adjustment with the inclusion of a beta‐blocking agent. Once an effective dose is reached, many centers allow an additional 1–2 weeks of stabilization before surgery, to facilitate intravascular volume repletion and reversal of catecholamine‐mediated organ dysfunction.

A high‐sodium diet with liberal fluid intake is commonly recommended after initiation of α‐adrenergic blockade to expand intravascular volume and reduce the risk of severe hypotension after tumor removal [31]. However, the degree of volume expansion should be individualized, as not all patients are markedly volume contracted and aggressive salt or fluid loading may be inappropriate in patients with heart failure, renal impairment, or catecholamine‐induced cardiomyopathy.

The Endocrine Society guideline recommends preoperative hemodynamic goals to achieve such as seated blood pressure < 130/80, seated heart rate of 60–70 beats per minute, upright blood pressure > 90 systolic, and upright heart rate of 70–80 [42]. These values should be interpreted as general hemodynamic ranges rather than a single target measurement, as many patients with pheochromocytoma exhibit intermittent or paroxysmal hypertension. Accordingly, blood pressure should be assessed repeatedly during the optimization period rather than relying on a single clinic reading. The Roizen criteria are available to establish how well the alpha‐1 adrenergic blockade is before surgery [47]. Although historically influential, the Roizen criteria were liberal, developed more than 4 decades ago and should primarily be viewed as a general framework rather than strict contemporary thresholds for perioperative preparation. A synopsis of preoperative hemodynamic goals is provided in Table 3.

**TABLE 3:** A synopsis of preoperative hemodynamic goals in patients with pheochromocytoma.

Endocrine Society guidelines [42]	Roizen criteria [47]
Seated BP of 120/80 mm·Hg	BP < 160/90 mm·Hg for 24 h before surgery
Standing SBP ≥ 90 mm·Hg	Orthostatic blood pressure > 80/45 mm·Hg
Heart rate 60–70 beats per minute while seated	Twelve‐lead electrocardiogram free of ST‐T changes for at least 1 week before surgery
Heart rate 70 to 80 beats per minute while standing	No more than one premature ventricular contraction every 5 min

Common adverse effects of alpha‐1 adrenergic blockers include orthostatic hypotension, with attendant fall risk, nasal congestion, and retrograde ejaculation in men [42]. Urapidil, although not commonly used in the USA, is used more frequently elsewhere, particularly in parts of Europe, and has been described as a perioperative antihypertensive option in pheochromocytoma. Mechanistically, it acts primarily as a selective peripheral postsynaptic alpha‐1 adrenergic antagonist and also has a central sympatholytic effect mediated through serotonin 5‐HT1A receptor agonism [48, 49]. Its role for intraoperative management is limited by its long half‐life compared to other alternatives.

It is important to note that beta‐adrenergic blockers should not be initially started in patients with pheochromocytoma due to the potential risk of hypertensive crisis due to unopposed alpha‐receptor activation via endogenous catecholamines [50, 51]. At least several weeks of alpha‐1 blockade must be ensured before beta blockers are initiated if needed (e.g., persistent tachycardia). However, routine beta‐adrenergic blockade is not required for most patients and should generally be reserved for those with persistent tachycardia after adequate alpha blockade. Avoiding unnecessary long‐term beta blockade may help avoid negative inotropic effects and reduce the risk of perioperative tachycardia associated with abrupt beta blocker withdrawal. This rule also applies to nonselective beta blocker labetalol, given its undesirable beta‐1 to alpha‐1 receptor ratio of approximately three to one orally, but 7 to 1 if given intravenously [52]. There have also been case reports of severe hypertension when labetalol was used as a primary agent [53].

Calcium channel blockers can either be added or used in place of the alpha‐1 inhibitor regimen. The outcome data comparing alpha‐1 adrenergic blockers is limited, but the use of calcium channel blockers instead of alpha‐1 adrenergic antagonists appears to be safe [54].

Another agent available for preoperative optimization in pheochromocytoma is metyrosine [50]. Metyrosine inhibits tyrosine hydroxylase, the rate‐limiting enzyme in catecholamine synthesis (see Figure 1). This medication may be particularly useful in patients who cannot tolerate standard antihypertensive therapy, in those in whom marked catecholamine release is anticipated, such as during tumor ablation or with large tumors, and in patients in whom difficult surgical dissection or resection is expected [55]. However, because metyrosine may cause significant adverse effects, including severe diarrhea, tremor, extrapyramidal reactions, and oculogyric crisis, and because abrupt discontinuation may precipitate pheochromocytoma crisis, many clinicians reserve its use for selected patients, particularly those symptomatic with unresectable disease.

Diuretics should be avoided during preoperative optimization in patients with pheochromocytoma, who are typically intravascularly volume depleted, unless a compelling indication exists, as their use may exacerbate hypovolemia and increase the risk of perioperative hypotension [42]. A summary of antihypertensive medications that can be used in the management of pheochromocytoma is presented in Table 4.

**TABLE 4:** A summary of antihypertensive medications that can be used in the preoperative management of pheochromocytoma.

Medication	Receptor affinity/mechanism of action	Dosing	Adverse reactions
Doxazosin	Reversible *α*1 adrenergic blocker	2–4 mg every 8–12 h	Orthostatic hypotension
Phenoxybenzamine	Irreversible nonselective α‐adrenergic blocker (α‐1 to α‐2 blockade ratio of 250:1)	10 mg every 8–12 h	Orthostatic hypotension, nasal congestion
Urapidil	Selective *α-*1 adrenergic blocker with additional central sympatholytic effect via 5‐HT1A receptor agonism	30–60 mg orally twice daily, titrated to blood pressure response	Orthostatic hypotension, dizziness, headache, nausea
Amlodipine	Calcium channel blocker	5–10 mg once a day	Edema, headaches
Metyrosine	Tyrosine hydroxylase inhibitor	250–500 mg every 6 h	Sedation, extrapyramidal reactions (e.g., rigidity, tremor, bradykinesia, akathisia, dystonia, oculogyric crisis), psychiatric symptoms (e.g., anxiety, depression), gastrointestinal upset (e.g., abdominal pain, nausea, vomiting, diarrhea)

From a practical standpoint, inadequate preoperative preparation often becomes most apparent in the operating room, where its hemodynamic consequences must be managed by the anesthesiologist. Despite this, anesthesiology involvement in preoperative optimization remains variable across healthcare systems because of structural and resource limitations, and pharmacologic titration is often directed primarily by endocrinology or surgical teams. This gap underscores the importance of early preanesthesia evaluation and close interdisciplinary communication. A cardiologist should also be considered an important member of the multidisciplinary team, not only in patients with overt cardiac complications, but also as a contributor to perioperative hemodynamic assessment and blood pressure management.

## 6. Intraoperative Considerations in Patients With Known Pheochromocytoma

Intraoperative management of a patient with pheochromocytoma requires careful planning and close attention to the dynamic nature of the operation. The attending anesthesiologist must know relevant presurgical diagnostic workup (e.g., complete blood count, metabolic panel, blood type, baseline 12‐lead electrocardiogram, echocardiography), preoperative optimization strategies of the respective patient, including the medications used, presence of target organ damage such as cardiovascular disease (e.g., congestive heart failure, history of stroke), chosen surgical approach, and the size and location of the tumor. For example, left‐sided adrenalectomies may require splenectomy, which carries a higher bleeding risk, but right‐sided tumors sit very close to the vena cava and may have aberrant vessels directly feeding it. Patients with a history of previous adrenalectomy or to undergo bilateral adrenalectomies may need stress dose steroid supplementation [41]. But supplementation should not be given until after tumor resection, as glucocorticoids have been reported to precipitate pheochromocytoma crisis in some patients [56]. The surgery is typically performed under general anesthesia and a regional analgesic technique to improve postoperative pain control if an open procedure is performed [57].

In addition to preoperative α‐adrenergic blockade and judicious salt/fluid loading, effective circulating volume should be optimized before induction and reassessed during tumor dissection. In the immediate intraoperative period, IV fluid administration should be individualized, with replacement guided by blood loss, hemodynamic assessment, and evidence of fluid responsiveness; small studies in pheochromocytoma suggest that stroke volume variation‐ or esophageal Doppler‐guided fluid therapy may help avoid excessive fluid administration [58, 59]. Dynamic indices such as pulse pressure variation and stroke volume variation should be interpreted cautiously because pneumoperitoneum and increased intra‐abdominal pressure may alter their performance; furthermore, although most pheochromocytomas are resected minimally invasively, selected patients require planned open adrenalectomy or conversion to an open approach, and opening the abdominal cavity itself substantially decreases these indices [42, 60, 61]. After adrenal vein ligation or tumor removal, hypotension should prompt reassessment of volume status; however, abrupt catecholamine withdrawal and vasoplegia may predominate, and vasopressors should not be delayed once appropriate volume replacement has been achieved [59].

It is important to anticipate and prepare patients who may do worse intraoperatively [3, 4, 62–64]. It is prudent to pay closer attention to patients with larger tumors, higher levels of free catecholamines, preoperative myocarditis, cardiomyopathy, and degree of preoperative hemodynamic control, in whom longer duration of surgery is anticipated, including reoperative surgery, female subjects, individuals with lower body mass, patients with coronary artery disease, subjects with baseline anemia, and patients with diabetes mellitus.

The operating surgeon may choose from several available resection options [42]. In most circumstances, the operation can be performed laparoscopically or robotically, given less pain, shorter hospitalization, and decreased blood loss compared to an open approach. Two common approaches include trans abdominal (known as Gagner), in which the patient is typically positioned in the lateral decubitus, and retroperitoneal (known as Walz), which is performed in a prone position [42]. Careful attention to patient positioning is essential to ensure optimal surgical exposure and avoid position‐related complications [65]. Open surgical resection may be necessary in patients with large tumors, invasive pheochromocytomas, or if significant bleeding occurs during laparoscopic or robotic techniques [42]. Partial (sparing adrenal cortex) adrenalectomy is a viable surgical option for pheochromocytoma in carefully selected patients, particularly those with hereditary syndromes or bilateral tumors in whom preservation of adrenal cortical function is desirable. Compared with total adrenalectomy, this approach may reduce the risk of permanent adrenal insufficiency and lifelong steroid dependence but is associated with a higher risk of local recurrence, necessitating careful patient selection and long‐term biochemical surveillance [42].

Anesthesiologists must also be aware of medications that can trigger the release of endogenous catecholamines to mitigate the risk of surgery‐related hemodynamic perturbations [66]. The medications to avoid include metoclopramide, droperidol, glucocorticoids (before tumor resection), glucagon, ketamine, succinylcholine, atracurium, and ephedrine, among others [66]. These medications can lead to exaggerated hemodynamic responses in patients with pheochromocytoma either via potentiation of catecholamine release or reduced adrenergic hormone metabolism. In addition, systemic glucocorticoids have been reported to precipitate pheochromocytoma crisis, likely through increased catecholamine synthesis and enhanced adrenergic responsiveness with several fatal outcomes [56, 67, 68]. For this reason, routine administration of intraoperative stress dose steroids should be avoided prior to tumor resection unless a strong compelling indication exists such as established adrenal insufficiency, or chronic glucocorticoid dependence [69, 70]. Medications such as dexmedetomidine, propofol, lidocaine, and rocuronium appear to be safe for use when needed. A summary of commonly used anesthetic medications that should generally be avoided in patients with pheochromocytoma is presented in Table 5.

**TABLE 5:** Intraoperative medications to avoid in pheochromocytoma.

Drug class	Agents to avoid	Rationale
Opioids	Morphine (high dose), meperidine	Histamine release, vasodilation, reflex sympathetic activation
Antiemetics/dopamine antagonists	Metoclopramide, droperidol, prochlorperazine	Dopamine receptor blockade may augment endogenous catecholamine release by removing inhibitory dopaminergic control of adrenal chromaffin cells
Gastrointestinal agents	Glucagon	Direct stimulation of catecholamine secretion
Glucocorticoids	Hydrocortisone, dexamethasone, methylprednisolone	Glucocorticoids may precipitate pheochromocytoma crisis through increased catecholamine synthesis and enhanced adrenergic responsiveness. This has resulted in several associated fatal outcomes
Intravenous anesthetics	Ketamine	Sympathomimetic effects
Volatile anesthetics	Desflurane	Rapid increases in anesthetic concentration may provoke sympathetic activation, resulting in acute tachycardia, hypertension, and increased circulating catecholamine levels. Isoflurane and sevoflurane are generally favored because they are less likely to induce sympathetic stimulation when titrated
Neuromuscular blockers	Pancuronium	Vagolytic and sympathomimetic effects
Adrenergic agonist	Ephedrine	Ephedrine relies on endogenous catecholamine release, resulting in an unpredictable hemodynamic response ranging from minimal effect to profound hypertension
Antihypertensives	Beta blockers	Unless used as an adjunct in adequately optimized patients, unopposed alpha‐adrenergic stimulation may exacerbate hypertension

The decision to administer preoperative anxiolysis should be tailored to an individual patient with some potential benefits, including improved tolerance of the preinduction period. Patients with pheochromocytoma are at increased risk for perioperative tachyarrhythmias and abrupt hemodynamic instability due to catecholamine surges during induction and tumor manipulation, as well as sudden catecholamine withdrawal following tumor ligation. Prophylactic placement of external defibrillation pads should be considered prior to induction of anesthesia, particularly in patients with large or biochemically active tumors, preexisting arrhythmias, or anticipated significant hemodynamic lability. This approach facilitates immediate cardioversion or defibrillation without interrupting surgical workflow during critical periods. Given its low risk and potential benefit, defibrillation pad placement may be considered part of a risk‐mitigation strategy in selected patients.

An invasive arterial catheter should be placed before induction of general anesthesia and laryngoscopy to permit beat‐to‐beat blood pressure monitoring during periods of anticipated hemodynamic instability. Because peripheral arterial pressure measurements may be unreliable during high‐catecholamine states, placement of a more central arterial catheter, such as femoral or axillary arterial access, should be considered [71–73]. Arterial access also facilitates frequent blood sampling for blood gas, glucose, electrolyte, hemoglobin, and acid–base assessment when clinically indicated. The need for central venous access should be individualized according to tumor size, catecholamine secretory phenotype, anticipated intraoperative and postoperative hemodynamic instability, baseline ventricular function, the likelihood of requiring vasoactive infusions, and the adequacy of peripheral venous access. Reliable large‐bore peripheral access is essential, and central venous access is particularly useful when multiple titratable vasoactive agents (e.g., clevidipine, sodium nitroprusside), vasopressors (e.g., norepinephrine, vasopressin), or inotropes are anticipated, or when peripheral access is inadequate. In patients with significant cardiovascular disease, reduced ventricular function, catecholamine‐induced cardiomyopathy, or complex volume/inotrope requirements, pulmonary artery catheterization or intraoperative transesophageal echocardiography may be considered to guide hemodynamic management. When airway assessment permits, induction should proceed in a controlled manner, allowing adequate time for anesthetic agents to achieve sufficient depth before laryngoscopy and tracheal intubation, thereby attenuating sympathetic stimulation and hypertensive surges. Short‐acting, titratable antihypertensive agents should be immediately available before induction and throughout tumor manipulation, with vasopressors also prepared for hypotension after adrenal vein ligation, tumor devascularization, or tumor removal.

Different stages of the operation are associated with distinct hemodynamic responses, such as hypertensive response during laryngoscopy and tumor manipulation, as well as hypotensive response after the isolation of the effluent adrenal vein or other aberrant veins [57]. Thus, close and dynamic intraoperative communication between the surgical and anesthesia teams is essential for the best possible outcome. It is essential to remember that even patients who were started on preoperative alpha‐adrenergic blockade and deemed appropriately optimized can still develop profound hypertensive response with intraoperative tumor manipulation [74].

Therefore, anesthesiologists must anticipate and be ready to address the intraoperative hemodynamic perturbations. If the clinical situation allows, one must ensure a deep level of sedation before airway instrumentation occurs. Antihypertensive medications, both in bolus forms as well as infusions, must be readily available. A list of first‐line antihypertensive medications available for intraoperative use is presented in Table 6, while second‐line blood pressure‐lowering agents are presented in Table 7 [75, 76].

**TABLE 6:** A summary of first‐line intravenous antihypertensive medications that can be used to manage hypertensive/tachycardic response in pheochromocytoma surgery.

Medication	Receptor affinity/mechanism of action	Dosing	Onset and duration of action	Adverse reactions
Sodium nitroprusside	Activation of cyclic guanylate monocyclase (GMP), vasodilator (arterial > venous)	0.25–2 mcg/kg/minute (avoid higher dosing because of the risk of cyanide toxicity)	Onset within 30 s to a minute. Duration of action 1–10 min	Hypotension, elevated intracranial pressure, cyanide (risk factors include higher dose, renal disease and liver disease), and thiocyanate toxicity
Nitroglycerin	Nitric oxide donor, GMP activation. Vasodilator (venous > arterial)	25–200 mcg as bolus. 5‐100 mcg/minute as continuous infusion	Onset within 5 min and duration of 5–10 min	Hypotension (avoid for 24 h if recent use of phosphodiesterase inhibitors such as sildenafil and for 48 h after the use of tadalafil), reflex tachycardia, methemoglobinemia
Clevidipine	Calcium channel blocker	1–32 mg/h continuous infusion	Onset within 2–3 min and duration of action 5–15 min (metabolized be blood esterases)	Reflex tachycardia, hypertriglyceridemia
Urapidil	Selective *α*1 adrenergic blocker with additional central sympatholytic effect via 5‐HT1A receptor agonism	Intravenous infusion, commonly 10–15 mg/hour with titration to hemodynamic response	Onset within about 5 min; duration approximately 2–8 h	Hypotension, dizziness, nausea, headache, fatigue
Esmolol	Selective β‐1 blocker	Bolus dosing 0.5 mg/kg. Continuous infusion 25‐200 mcg/kg/minute	Onset within 1–2 min and duration of action 10–30 min (metabolized by red blood cell esterases, duration may be prolonged in anemia)	Bradycardia, hypertensive response in patients suboptimally treated with alpha‐1 adrenergic blockers is possible, cardiogenic shock
Magnesium^∗^	Vasodilation via catecholamine release inhibition and endogenous calcium antagonist	Loading dose of 50 mg/kg over 30 min followed by 15 mg/kg/hour	Onset within 1–2 min and duration depends on several factors such as renal dysfunction	Potentiation of neuromuscular blockade, bradycardia, hypotension, cardiac arrest

^∗^Magnesium is not classically considered to be the antihypertensive agent.

**TABLE 7:** A summary of second‐line intravenous antihypertensive medications that can be used to manage hypertensive response in pheochromocytoma surgery.

Medication	Receptor affinity/mechanism of action	Dosing	Onset and duration of action	Adverse reactions
Phentolamine	α‐1 adrenergic blocker	5–15 mg bolus every 15 min as needed. Continuous infusion 1‐6 mcg/kg/minute	Onset within 1–2 min and duration of 10–30 min	Hypotension, reflex tachycardia
Nicardipine	Calcium channel blocker	5–15 mg/hour continuous infusion	Onset within 5–15 min and duration of action approximately 4–6 h (can be prolonged in liver disease)	Reflex tachycardia, venous thrombophlebitis
Verapamil	Nondihydropyridine calcium channel blocker; inhibits L‐type calcium channels with predominant effects on atrioventricular nodal conduction, heart rate reduction, and modest arterial vasodilation	Bolus dosing 5–10 mg IV administered over 2–3 min; may repeat after 15–30 min if needed. Continuous infusion 5–20 mg/hour	Onset within 1–3 min. Duration of action approximately 10–20 min (longer with repeated dosing or infusion)	Bradycardia, atrioventricular block, hypotension, negative inotropy, cardiogenic shock in patients with systolic ventricular dysfunction
Diltiazem	Similar to verapamil	Bolus dosing 0.25 mg/kg IV over 2 min; may repeat with 0.35 mg/kg after 15 min if needed. Continuous infusion 5–15 mg/hour	Onset within 2–5 min. Duration of action approximately 30–60 min	Similar to verapamil
Labetalol	Nonselective β‐1 and α‐1 blocker (ratio of β‐1 to α‐1 is 7:1 for IV formulation)	Bolus dosing 5–20 mg every 15 min as needed. Continuous infusion 0.5–2 mg/min	Onset within 5–10 min and duration of 3–6 h	Bradycardia, bronchospasm, exaggerated hypertensive response in patients suboptimally treated with alpha‐1 adrenergic blockers is possible, cardiogenic shock
Metoprolol	Selective β‐1 blocker	Bolus dosing 2.5–5 mg every 10–15 min as needed	Onset within 5–10 min and duration of action 4–6 h	Bradycardia, hypertensive response in patients suboptimally treated with alpha‐1 adrenergic blockers is possible, cardiogenic shock
Hydralazine	Direct arteriolar vasodilator	Bolus dosing 5–20 mg	Onset within 15–20 min and duration of 3–4 h	Unpredictable blood pressure response (from limited efficacy to pronounced hypotension) is possible, reflex tachycardia

After the adrenal vein is clamped, one must anticipate hypotension. The sudden loss of catecholamine release, adrenergic receptor downregulation, vena cava compression, blood loss, and preoperative antihypertensive therapy are all potential mechanisms [51]. In addition to close intraoperative communication, management includes intravascular fluid repletion (including blood products if bleeding) and vasoactive medication administration. Vasopressors with nonadrenergic mechanisms of action, such as vasopressin and angiotensin, should be readily available for managing refractory hypotension and vasoplegia [55, 77]. In some patients, particularly those with preexisting cardiomyopathy or catecholamine‐induced myocardial dysfunction, hypotension may also reflect impaired myocardial contractility rather than isolated vasoplegia [78]. In such cases, inotropic support may be required in addition to vasopressors and volume resuscitation. Advanced hemodynamic monitoring, including pulmonary arterial catheterization with continuous cardiac output and continuous mixed venous O_2_ saturation (SvO_2_) can guide inotrope therapy. Dynamic variables such as pulse pressure variation may also be useful. Transesophageal echocardiography may be used, but not ideal in patients in the lateral decubitus position and may be difficult to perform in that setting. Furthermore, one must anticipate the potential for hypoglycemia after adrenal vein isolation, which is unpredictable and can be unrelated to cardiovascular events.

## 7. Intraoperative Management of Patients With Previously Undiagnosed Pheochromocytoma

Several aspects of pheochromocytoma, such as its rarity, protean and nonspecific clinical manifestations, or the presence of no to minimal symptoms, pose unique challenges to its timely diagnosis and management [8, 79]. Thus, some patients will present with no prior outpatient medical care or workup. Even in developed countries, the preoperative diagnosis of pheochromocytoma can be missed even in patients presenting for elective adrenal resection surgery [10]. It is interesting to note that some of the patients with previously undiagnosed pheochromocytoma did well intraoperatively for unrelated surgery only to become hypertensive and symptomatic during their recovery period, which triggered further workup [80].

Kirshtein et al. retrospectively analyzed 126 patients who underwent adrenalectomy within the University of Ottawa healthcare system [81]. Adrenal incidentaloma was the primary indication for surgery in 45 patients, of whom 13 were ultimately diagnosed with biochemically and pathologically confirmed pheochromocytoma; notably, 7 of these 13 patients had no prior history of hypertension. Despite preoperative treatment with alpha‐1 adrenergic blockade followed by beta‐adrenergic blockers, significant intraoperative hemodynamic instability occurred in 7 patients. Importantly, three pheochromocytomas were detected incidentally during imaging performed for motor vehicle‐related trauma; none of these patients had hypertension at the time of presentation, and two had no antecedent history of hypertension. In two cases, trauma‐related orthopedic surgery was deferred until appropriate pharmacologic optimization and definitive adrenalectomy were completed. In the third case, traumatic injuries were managed nonoperatively, allowing for medical optimization prior to subsequent tumor resection.

Some potential clues to suggest that the patient may have undiagnosed pheochromocytoma and alert the anesthesiologist include an exaggerated response to laryngoscopy and intubation despite what seems to be adequate induction doses of anesthetic agents and the plane of sedation, hypertensive response to abdominal insufflation and unrelated abdominal surgery, potentially difficult to control intraoperative hypertension, uncontrolled arrhythmias, paradoxical hypertensive response or worsening hypertension after beta blocker administration, and hypertensive response to metoclopramide, droperidol, or glucocorticoids.

Upon suspicion of an intraoperative pheochromocytoma crisis, the anesthesiologist should call for help and immediately deepen anesthesia, ensure adequate analgesia, and eliminate surgical stimulation if feasible. Rapid blood pressure control should be achieved using short‐acting intravenous vasodilators such as sodium nitroprusside, nicardipine, or clevidipine. In patients with significant tachycardia, nondihydropyridine calcium channel blockers (e.g., verapamil or diltiazem) may be used for rate control. However, β‐blockers should generally be avoided in the acute management of untreated pheochromocytoma, although rare exceptions have been reported [82]. In one case report, esmolol was used as initial therapy in a critically ill patient with an epinephrine‐secreting pheochromocytoma, severe tachyarrhythmias, and coexisting coronary artery disease, allowing rapid titration because of its short elimination half‐life [82]. The authors emphasized that this approach should be reserved for carefully selected patients with intensive hemodynamic monitoring and should not replace the usual principle of α‐blockade before β‐blockade.

Importantly, in patients with underlying systolic ventricular dysfunction, which can be assessed intraoperatively using transthoracic (when surgical draping permits) or transesophageal echocardiography, nondihydropyridine calcium channel blockers and beta blockers may precipitate cardiogenic shock and should be avoided. A summary of commonly available intravenous antihypertensives for the management of intraoperative hypertensive crisis is presented in Table 6 [8]. Continuous invasive arterial blood pressure monitoring should be established if not already in place, and close communication with the surgical team is essential to determine whether the procedure should be aborted or limited to life‐ or organ‐threatening pathology. In most cases, surgery should be aborted.

## 8. Postoperative Management

The decision of whether the postoperative patient gets admitted to the intensive care unit (ICU) versus the down care floor should consider both patient‐specific and institutional practices and guidelines. For example, preemptive ICU admission should be considered in patients who are deemed to be at high risk for complications, such as with large tumors, elevated levels of free catecholamines, and a degree of preoperative hemodynamic control, in whom longer surgery is expected, including reoperative surgery, female patients, individuals with lower body mass, patients with myocarditis, cardiomyopathy, or coexisting coronary artery disease, subjects with baseline anemia, and patients with diabetes mellitus [3, 4, 62–64]. Additionally, patients with major intraoperative complications (e.g., significant bleeding), hypotension requiring vasopressor therapy, and hypoglycemia requiring intensive correction must be transferred to the ICU for postoperative care.

Common complications of adrenal resection for patients with pheochromocytoma include residual hypertension, hypotension, hypoglycemia, acute kidney injury, arrhythmias, and acute cardiac and cerebrovascular events [83]. Below, we will discuss some key and common postoperative complications of pheochromocytoma resection and their management. The reader is referred to a well‐written review article focused on the topic of postoperative pheochromocytoma care [82].

Residual postoperative hypertension after adrenal resection may signify incomplete tumor resection, inadequate analgesia and sedation, medication error (e.g., inadvertent vasopressor administration), hypervolemia, and coexistent essential hypertension. It is important to achieve target blood pressure to decrease the risk of perioperative stroke, cardiac events, and postoperative bleeding by targeting what appears to be the underlying cause of this elevated blood pressure. Patients who are initially hypertensive may still develop hypotension later in their postoperative course.

Postoperative hypotension is another important complication that may occur after surgery. Common causes include suboptimal preoperative or intraoperative intravascular volume expansion with persistent hypovolemia, bleeding, adrenergic receptor desensitization, residual effects of adrenergic blocking medications, and procedure‐related complications such as pneumothorax after central venous catheter placement. Management should be directed at the underlying cause and generally includes intravascular volume resuscitation, administration of blood products when bleeding is present, and vasopressor support when needed. After hypovolemia has been excluded, some patients may exhibit a combination of vasoplegia and reduced inotropic state, in which case assessment of cardiac function and appropriate use of inotropic agents, such as epinephrine, should be considered.

Postoperative hypoglycemia may occur because of the abrupt decline in catecholamine levels after adrenal resection leads to unopposed insulin activity and other factors. This risk may be further increased because some patients with pheochromocytoma have elevated insulin levels [84]. Management is supportive and centers on frequent blood glucose monitoring and maintenance of euglycemia. Prophylactic administration of glucose‐containing solutions, such as 5% dextrose in water or 5% dextrose in lactated Ringer solution, is indicated postoperatively until the patient’s glycemic pattern has been established and higher concentrations of dextrose‐containing solutions (such as D_10_ or D_20_) may be necessary in some patients. This phenomenon usually resolves in 24–48 h postoperatively.

## 9. Conclusion

Pheochromocytoma is a rare neuroendocrine tumor with nonspecific and protean clinical presentations. If not recognized and treated, it is associated with the development of target organ damage and complications such as cardiac ischemia, cardiomyopathy, cerebrovascular accidents, and diabetes mellitus, among many others. The tenets of pheochromocytoma diagnosis include biochemical evidence of catecholamine excess followed by imaging localization of the tumor. Patients with newly diagnosed pheochromocytoma should be seen by multidisciplinary perioperative teams, including anesthesiologists, surgeons, and endocrinologists.

Perioperative antihypertensive therapy should be instituted prior to adrenal resection to allow recovery from organ injury resulting from catecholamine excess and to mitigate intraoperative hypertensive events. Organ recovery should inform appropriate timing for surgery. The anesthesiologist must be prepared to manage both intraoperative hypertension and hypotension and glycemic disturbances, among other possible complications. Patients with undiagnosed pheochromocytoma who present for unrelated surgery represent a high‐risk group. Once suspected, management should focus on immediate hemodynamic stabilization, minimization of surgical stimulation, and close communication with the surgical team regarding whether the procedure should be aborted, limited to life‐ or organ‐threatening pathology, or in rare cases continued based on the clinical circumstances. In most cases, nonessential surgery should be aborted. Postoperative level of care should be based on intraoperative patient course and institutional protocol and guidelines.

## Author Contributions

A.E.M. and N.B. contributed equally to the conception and design of the review, literature review, drafting of the manuscript, and critical revision for important intellectual content.

All authors contributed equally to the design, preparation, and editing of this manuscript.

## Funding

The authors declare that no funds, grants, or other support were received during the preparation of this manuscript.

## Disclosure

All authors reviewed and approved the final manuscript and take responsibility for all aspects of the reliability and freedom from bias of the presented data and their discussed interpretation.

## Conflicts of Interest

The authors declare no conflicts of interest.

## Data Availability

The data supporting the findings of this study are derived from previously published articles, which are cited in the reference list. No new data were generated.
